# Research on the effect of LAMP1 in the development and progression of ccRCC and its potential mechanism with LC3C-mediated autophagy

**DOI:** 10.3389/fimmu.2024.1494005

**Published:** 2024-11-28

**Authors:** Xiongbao Wang, Liang Fang, Lixiang Xiao, Guangxin Zhong, Minghao Han, Bingshen Wang, Juchao Ren, Yuanwei Zang

**Affiliations:** ^1^ Department of Urology, Qilu Hospital of Shandong University, Jinan, Shandong, China; ^2^ Center for Reproductive Medicine, Department of Obstetrics and Gynecology, Qilu Hospital of Shandong University, Jinan, Shandong, China; ^3^ Laboratory of Basic Medical Sciences, Qilu Hospital of Shandong University, Jinan, Shandong, China; ^4^ Cheeloo College of Medicine, Shandong University, Jinan, Shandong, China; ^5^ Department of Urology, School of Clinical Medicine, Beijing Tsinghua Changgung Hospital, Tsinghua University, Beijing, China

**Keywords:** LAMP1, renal cancer, biomarkers, bioinformatics, prognosis

## Abstract

**Background:**

Lysomembrane-associated protein 1 (LAMP1), known to exhibit differential expression in various tumor types and play a crucial role in the development of tumors. Clear cell Renal Cell Carcinoma (ccRCC) is still the most common pathological type of renal carcinoma with poor prognosis. However, the expression of LAMP1 and its underlying molecular mechanism with ccRCC remain elusive.

**Methods:**

Firstly, the expression of LAMP1 in ccRCC and its clinical significance were analyzed using various databases. Next, Weston Blot was performed to detect the expression of LAMP1 protein in cancer tissues and adjacent tissues from 60 pairs of clinical ccRCC patients. The correlation between LAMP1 expression and different clinical indicators as well as the relationship with patient prognosis was analyzed. Furthermore, molecular cell biology experiments were conducted to validate the effects of LAMP1 gene expression on cell proliferation, invasion and migration. Additionally, we investigated the impact of VHL, a key gene in renal cancer, and LC3C, an autophagy-related gene, on LAMP1 expression through molecular biology experiments to elucidate the potential underlying mechanism.

**Results:**

Bioinformatics analysis revealed significant underexpression of LAMP1 in ccRCC (P<0.001), which correlated with poorer prognosis. In multivariate survival analysis, LAMP1 emerged as an independent prognostic marker for overall survival(OS)(P<0.05). Analysis of cancer and paracancer tissue samples from ccRCC patients demonstrated significantly lower levels of LAMP1 in tumors compared to paracancerous tissues (P<0.001), confirming its prognostic impact. Cell functionality experiment revealed that elevated LAMP1 inhibited cell proliferation, migration, and invasion. LAMP1 expression remained unchanged during autophagy modulation but decreased with LC3C knockdown and vice versa. Notably, VHL(+) cells expressed less LAMP1 than VHL(-) cells.

**Conclusions:**

These findings indicate that low expression levels of LAMP1 is associated with poor prognosis in ccRCC. Therefore, LAMP1 emerges as a novel biomarker associated with the diagnosis and prognosis of renal cancer. Furthermore, we have also described the potential mechanism of action of LAMP1 in renal cancer. LAMP1 is a promising target for the treatment of ccRCC.

## 1 Introduction

Renal cell carcinoma (RCC) is a highly prevalent neoplasm of the urinary system, exhibiting an escalating global incidence and mortality rate (1). Clear cell Renal Cell Carcinoma (ccRCC), accounting for approximately 75% of all cases, represents the predominant pathological type within RCC and serves as the primary cause of RCC-associated deaths (2). With the improvement of imaging and ultrasound technology, new advances have been made in the diagnosis of renal cancer (3, 4), yet still 30% of kidney cancer patients present with metastatic disease at the primary diagnosis (5). Moreover, the 5-year survival rate for advanced renal cancer patients stands at a mere 11.2% (6). Given its high heterogeneity, conventional radiotherapy exhibits limited efficacy against RCC. Consequently, molecular targeted therapy emerges as a crucial treatment option due to its ability to precisely target key molecular pathways involved in tumor growth and metastasis. Therefore, it becomes imperative and indispensable to identify reliable biomarkers while exploring potential molecular mechanisms for early diagnosis and precision treatment of RCC.

Lysosomal membrane-associated proteins (LAMPS) are a family of glycoproteins predominantly localized on lysosomal membranes. LAMP1 and LAMP2, the key members of this protein family, are ubiquitously expressed in mammals. They constitute the major constituents of lysosomal membranes, accounting for approximately 50% of all membrane proteins (7). LAMPs play crucial roles in various cellular processes including phagocytosis, autophagy, lipid transport, and senescence (8). In addition to their essential role in maintaining lysosomal function, LAMP1 is also implicated in autophagy regulation as well as tumor cell metastasis and invasion, thereby influencing tumor progression. However, the specific involvement of LAMP1 in renal carcinoma remains unexplored.

Autophagy is a catabolic process in which intracellular components are sequestered within double-membrane vesicles, known as autophagosomes, that subsequently merge with lysosomes to form autolysosomes. Within this compartment, the contents undergo degradation and recycling into the cytoplasm. Autophagy is tightly regulated at the cellular level and holds significant implications in tumorigenesis (9). Selective regulation of autophagy occurs in tumor cells. The three homologs of microtubule-associated protein 1 light chain 3 (LC3A, LC3B, and LC3C) along with members of GABA-type A receptor-associated proteins (GABARAPs) constitute crucial components of the autophagy regulatory pathway. Autophagy mediated by the classical regulatory pathway involving LC3B plays a pivotal role in ccRCC progression and is inhibited by VHL (10, 11). Studies have demonstrated that LC3C-mediated autophagy selectively governs migration and invasion of tumor cells stimulated by Met tyrosine kinase (RTK) and hepatocyte growth factor (HGF) (12). However, the mechanism underlying LC3C’s involvement in renal cancer remains elusive, thereby offering a novel avenue for investigating autophagy in renal cancer.

In this study, through our analysis, we have identified that LAMP1 exhibits low expression in ccRCC, and this decreased expression of LAMP1 is generally associated with a poorer prognosis for patients. Functional studies on cells have demonstrated the inhibitory effects of LAMP1 on renal cancer cell proliferation, migration, and invasion. Furthermore, it is suggested that the tumor-suppressive role of LAMP1 may be mediated by LC3C-induced autophagy. Therefore, our study represents the first validation of the novel potential biomarker LAMP1 in ccRCC, which holds promise for its application in diagnosing and prognosticating renal cancer.

## 2 Materials and methods

### 2.1 Differential expression analysis

The TIMER database (https://cistrome.shinyapps.io/timer/) is a website facilitating comprehensive analysis of diverse cancer gene expression patterns and the infiltration of tumor immune cells (13). It also enables individual gene expression analysis across various carcinomas. The TCGA database (https://tcga-data.nci.nih.gov/tcga/) encompasses extensive information on human cancers, including genomic variations, mRNA and miRNA expressions, as well as methylation profiles. For this study, two datasets from the TCGA database were utilized: RNA-seq transcriptome data along with corresponding clinical data from ccRCC samples. The UALCAN database(http://ualcan.path.uab.edu/index.html) provides in-depth RNA-SEQ expression analysis derived from the TCGA database and facilitates comparative assessment of protein expression disparities between tumor tissues and normal tissues (13).

### 2.2 Immunoinfiltration analysis

By employing the single sample gene enrichment analysis (ssGSEA) algorithm, we conducted a quantitative assessment of the relative levels of tumor invasion for 24 immune cell types. The correlation between LAMP1 and immune cell infiltration levels, as well as the association between immune cell infiltration and different expression groups of LAMP1, were investigated using Wilcoxon rank sum test and Pearson correlation analysis.

### 2.3 Survival analysis and prognostic model

The correlation between clinicopathological features and overall survival (OS), progression-free interval (PFI), and disease-specific survival (DSS) of patients in the TCGA database was analyzed using COX regression and Kaplan-Meier methods. Based on the results of COX regression, a nomogram was developed using independent prognostic factors obtained from multivariate analysis to predict individual 1-year, 3-year, and 5-year survival rates.

### 2.4 Clinical samples

Cancerous tissues and adjacent tissues (distance from the edge of tumor lesion> 2 cm) were collected from patients with ccRCC, confirmed by routine postoperative pathology after renal resection in the Department of Urology at our hospital between May 2016 and December 2020. A total of 60 pairs of cancerous and paracancerous tissues were obtained through meticulous screening. Prior to surgery, none of the patients received targeted drugs or immunosuppressive medications. All specimens excised during the operation were rinsed with 0.9% sodium chloride solution and promptly preserved in liquid nitrogen for storage purposes. The tumor size was determined based on measurements taken from postoperative pathological specimens, using the longest tumor diameter as a reference standard. TNM staging and nuclear grading were performed according to the AJCC’s 8th edition guidelines and WHO/ISUP grading system, respectively. Prior to surgery, all patients enrolled in the study provided written informed consent, which was duly approved and reviewed by the Ethics Committee at Qilu Hospital, Shandong University. A total of sixty patients were followed up via telephone. Patients exhibiting local tumor progression, recurrence, distant metastasis prior to surgery or new metastasis after surgery during follow-up were classified as experiencing tumor progression.

### 2.5 Extraction of histopin and Western blot

The collected patient specimen tissue is retrieved from the liquid nitrogen and sectioned to an appropriate size. Following thorough grinding, RIPA buffer supplemented with Phenylmethanesulfonyl fluoride and phosphatase inhibitors was added, facilitating total protein extraction through further grinding. Subsequently, the proteins were separated via 10% SDS-polyacrylamide gel electrophoresis and transferred onto a polyvinylidene fluoride membrane using electrotransfer. The membrane was then obtained and subjected to blocking with a solution containing 5% skim milk for one hour. It was subsequently incubated overnight at 4°C with the primary antibody, followed by an additional hour at room temperature with the secondary antibody. These bands were detected utilizing Pro-lighting in conjunction with ImageJ semi-quantitative analysis HRP images.

### 2.6 Cell culture

The human ccRCC cell lines A498 and 786-O were obtained from ATCC (Manassas, VA, USA). A498 cells were cultured in Dulbecco’s Modified Eagle’s Medium supplemented with 10% fetal bovine serum (FBS: Gibco, Grand Island, NY, USA). The 786-O cell line was cultured in Roswell Park Memorial Institute 1640 medium containing 10% fetal bovine serum. Both cell lines were maintained in a culture medium supplemented with 100 U/mL penicillin and 100μg/mL streptomycin. All cells were incubated at 37°C and 5% CO2 in a humidified incubator.

### 2.7 Virus transfection

Flag-tagged lentiviral vectors encoding human LAMP1 (LV5-human Lamp1 + 3XFlag) and empty vectors (LV5NC) were generated in T293 cells (GenePharma, Shanghai, China). Stable expression of LAMP1 was established in 786O and A498 human renal carcinoma cell lines by transfecting them with LAMP1 lentivirus at a confluency of 20%-30% using Lipofectamine 2000 reagent (Thermo Fisher Scientific). The stable transfection strains were selected using purinomycin at a concentration of 2μg/ml (Sigma-Aldrich, St. Louis, MO, USA), which based on our previous experiments which found that 2μg/ml of puromycin can effectively kill non-transfected cells while allowing transfected cells to survive and proliferate. And the efficiency of cell infection was evaluated by fluorescence microscopy after 72 hours. The lentivirus-transfected cells were prepared for subsequent experiments.

### 2.8 siRNA treatment

The chemically modified siRNAs targeting LAMP1 were used in conjunction with control siRNA purchased from Thermo Fisher Technologies. The specific sequence for LAMP1 siRNA1 was 5 ‘-CAGGGCAGAUAUAGAUAAATT-3’. while the sequence for LAMP1 siRNA2 was 5 ‘-CAGAGACCCUGCCUUUAAATT-3’. Cells were transfected with 100 nM of these siRNAs using Lipofectamine 2000 (Thermo Fisher Scientific) following the manufacturer’s instructions.

### 2.9 CCK-8 assay

The cell proliferation rate was assessed using the CCK-8 assay. The following cell lines were seeded in logarithmic growth phase onto a 96-well plate, each well containing a density of 2000 cells: 786-O/NC (786-O cells transfected with an empty vector), A498/NC (A498 cells transfected with an empty vector), 786-O/LAMP1 (786-O cells transfected with a LAMP1 overexpression vector), A498/LAMP1 (A498 cells transfected with a LAMP1 overexpression vector), 786-O/LAMP1+siRNA (786-O cells first transfected with a LAMP1 overexpression vector and subsequently treated with siRNA to knockdown LAMP1 expression), and A498/LAMP1+siRNA (A498 cells first transfected with a LAMP1 overexpression vector and subsequently treated with siRNA to knockdown LAMP1 expression). After incubation for 24 h, 48 h, and 72 h at 37°C, each well was supplemented with 10 μl of CCK-8 reagent and further incubated for an hour. Subsequently, the absorbance values at a wavelength of 450 nm were measured using a Microplate Reader (Bio-Rad, Hercules, CA).

### 2.10 Migration and invasion assays

The migration and intrusion tests were performed using the transwell system (Corning Costar, Lowell, MA, USA), as previously described. In the migration experiment, 10^6^ 786-O and A498 cells were suspended in 200 μl of serum-free medium and added to the upper compartment. Subsequently, 600 μl of complete medium was added to the lower chamber. After incubation for either 24 or 36 hours, cells that had migrated through the membrane were fixed with 4% paraformaldehyde for 30 minutes and then stained with 0.5% crystal violet for another 30 minutes. For the invasion test, a similar procedure was followed except that Matrigel (BD Biosciences, USA) was used to coat the membrane. Five random visual fields were selected under a microscope for observation and cell counting.

### 2.11 Wound-healing assay

First, utilize a marker to create 3-5 uniform straight lines on the underside of the 6-well plate, and evenly seed different cell types in the logarithmic growth phase at a density of 2×10^5^ cells per well into the designated wells; Once all wells are filled with cells, draw a straight line across the plate using the tip of a 200ul pipette and remove any isolated cells with phosphate buffered saline (PBS).

### 2.12 Colony formation assay

The cell suspension was prepared and inoculated into 6-well plates at a density of approximately 10^3^ cells per well. Following a 14-day incubation period, the cells were fixed with 75% methanol and stained with 0.1% crystal violet. After air-drying, the colonies containing more than 50 cells per well were visually documented and quantified.

### 2.13 Statistical analysis

The statistical analysis was performed using SPSS statistical software and Graphpad Prism8.4.3. The Mann-Whitney test was employed to analyze the levels of LAMP1 mRNA in different clinicopathological parameters of ccRCC. Pearson Chi-square test was utilized to examine the correlation between LAMP1 expression level and clinicopathological parameters of ccRCC. ROC curve analysis was conducted to evaluate the ability of LAMP1 expression levels in distinguishing ccRCC patients, obtaining the area under the curve (AUC). Kaplan-Meier curve analysis was applied to investigate the relationship between LAMP1 expression level and overall survival as well as progression-free survival among ccRCC patients. Each dataset is presented as mean ± standard deviation, while a significance level of P < 0.05 is considered statistically significant.

## 3 Results

### 3.1 Expression of LAMP1 in ccRCC and other tumors

**Figure 1:**
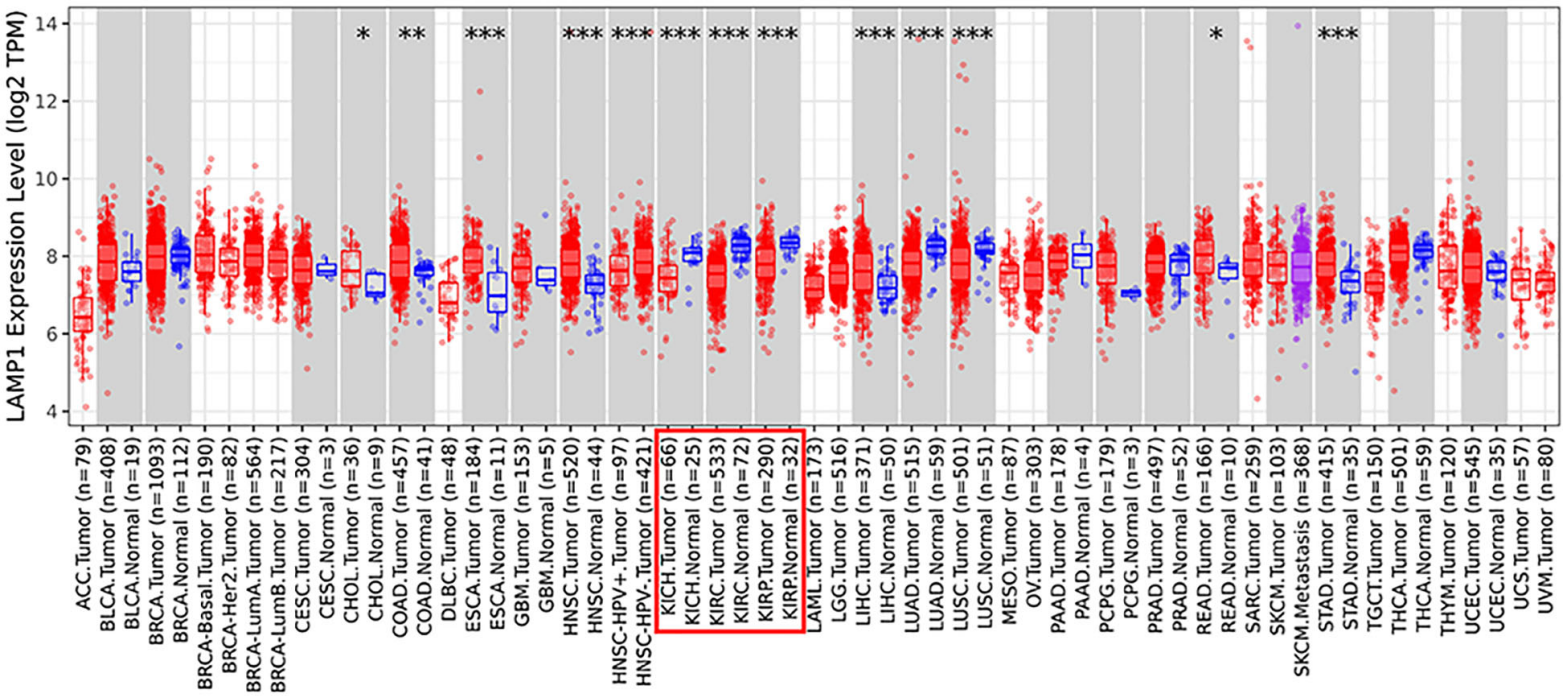
Expression of LAMP1 in different tumor types in TIMER2.0 database (*P<0.05, **P<0.01, ***P<0.001).

**Figure 2:**
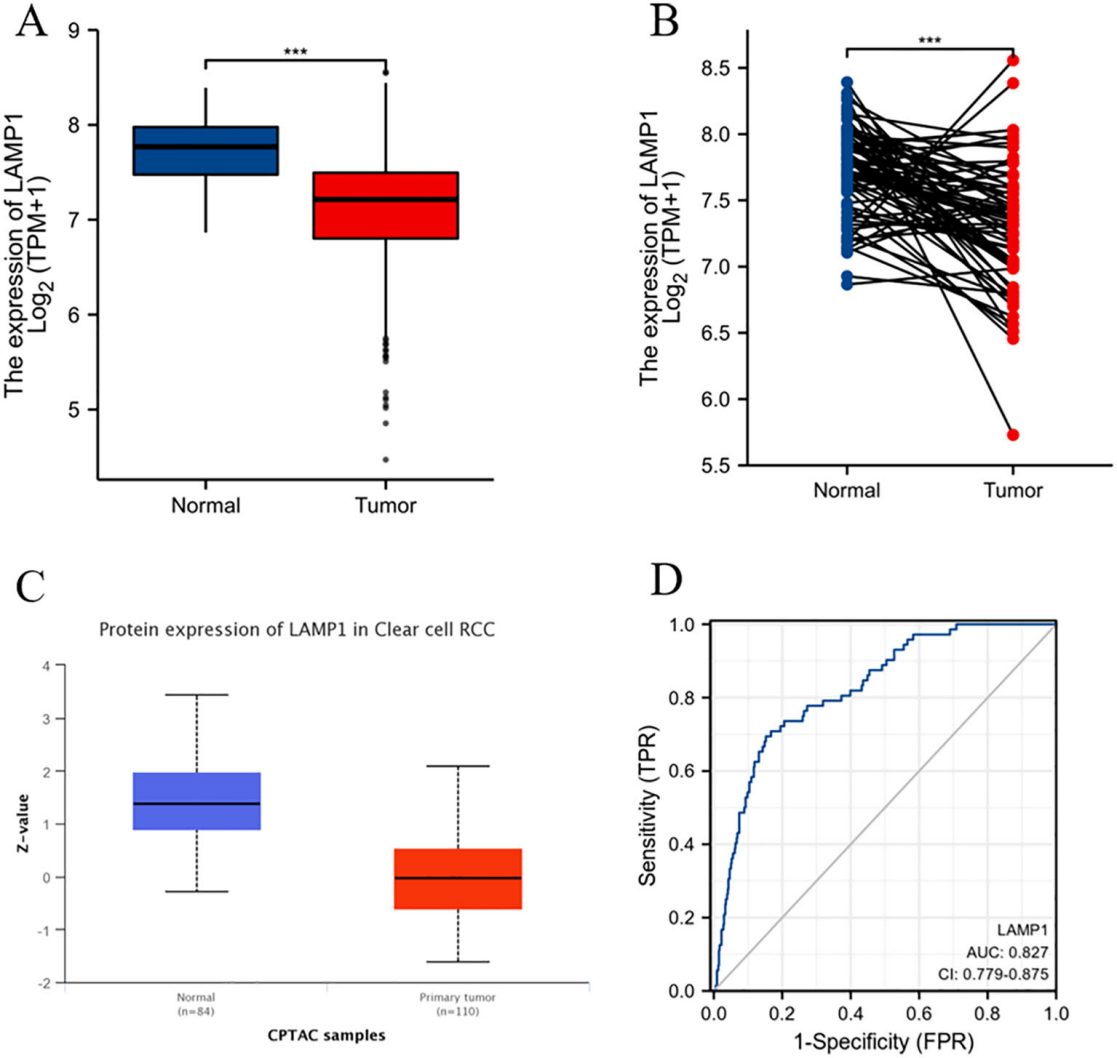
mRNA and protein expression levels of LAMP1 in ccRCC and ROC curve analysis **(A)** LAMP1 mRNA expression levels in ccRCC tissues (n=539) and normal tissues (n=72); **(B)** LAMP1 mRNA expression levels in ccRCC tissues (n=72) and its paired tissues (n=72); **(C)** LAMP1 protein expression level in UNCLAN database; **(D)** ROC curve to assess the differentiation of LAMP1 between tumor and normal tissue (***P<0.001).

The analysis using the TIMER2.0 database revealed differential expression levels of LAMP1 mRNA across various tumor types(**Figure 1**). Notably, LAMP1 was significantly downregulated in renal cancers, including Kidney Renal Clear Cell Carcinoma (KIRC), Kidney Chromophobe (KICH) and Kidney Renal Papillary Cell Carcinoma (KIRP), as well as lung cancers such as lung adenocarcinoma (LUAD) and lung squamous cell carcinoma (LUSC). Conversely, it exhibited high expression in cholangiocarcinoma (CHOL), colon cancer (COAD), rectal adenocarcinoma (READ), esophageal cancer (ESCA), gastric cancer (STAD), hepatocellular carcinoma (LIHC) and head and neck squamous cell carcinoma (HNSC). Next, we specifically analyzed the expression of LAMP1 in ccRCC (**Figure 2**). A comprehensive analysis of the RNA sequencing data from 539 ccRCC patients and 72 normal tissues sourced from the TCGA database revealed a significant decrease in LAMP1 mRNA expression within tumor tissues, both in unpaired samples (n = 611) and paired samples (n = 72) (**Figures 2A, B**, P<0.001). Furthermore, we utilized the UALCAN database to analyze the protein level of LAMP1 in ccRCC tissues compared to normal tissues. The results demonstrated a lower abundance of LAMP1 protein in ccRCC tissues than that observed in normal tissues(**Figure 2C**, P<0.01). Additionally, ROC curves were employed to assess the discriminatory potential of LAMP1 expression between cancerous and adjacent non-cancerous tissues (**Figure 2D**). The findings indicated an area under the curve(AUC) value for LAMP1 at 0.827,suggesting its potential utility as a discriminator for ccRCC.

### 3.2 Association between LAMP1 expression and clinical features of ccRCC

In order to elucidate the role and significance of LAMP1 expression, a total of 539 cases comprising LAMP1 expression data and comprehensive clinical information of ccRCC patients were retrieved from the TCGA database. The cohort consisted of 186 females (34.5%) and 353 males (65.5%), with an average age of 61 years (range: 52-70 years). Detailed patient information is provided in **Supplementary Table S1**. As depicted in **Supplementary Figures S1A–E**, there was a significant correlation between differential expression and T staging of LAMP1 (T1-2 vs. T3-4, P<0.01), clinicopathological stage (Stage I-II vs. Stage III-IV, P<0.001), histological grade (G1-2 vs G3-4, P<0.01), sex (female vs male, P<0.05), as well as race (Asian & Black or African American vs White, P<0.001). Notably, lower levels of LAMP1 expression were observed in tumors with higher stages and grades, and the expression levels were even lower in males and whites; however, its expression level remained independent of M and N stages, age, and tumor location within the left or right kidney according to **Supplementary Figures S1F–I** findings. Univariate logistic regression analysis revealed that categorical assessment of LAMP1 expression was associated with poor prognostic clinicopathological features as shown in **Table 1**. Level of LAMP1 expression in ccRCC was associated with T stage (OR=0.569, T1-2 vs. T3-T4), M grade (OR=0.570, M0 vs. M1), clinicopathologic stage (OR=0.487, Stage I-II vs. Stage III-IV) and histological grade (OR=0.610, G1-2 vs. G3-4) were negatively correlated (P<0.05). However, it was not related to N stage, gender, age and other factors (all P values> 0.05).

**Table 1:** Logistic regression analysis of correlation between LAMP1 expression and clinical features.

Characteristics	Total (N)	Odds Ratio (OR)	*P* value
Gender (Male vs. Female)	539	0.724 (0.506-1.033)	0.075
Age (>60 vs. <=60)	539	0.881 (0.628-1.236)	0.464
T stage (T3&T4 vs. T1&T2)	539	0.569 (0.397-0.813)	0.002
N stage (N1 vs. N0)	257	0.759 (0.263-2.101)	0.595
M stage (M1 vs. M0)	506	0.570 (0.344-0.931)	0.027
Pathologic stage (Stage III & Stage IV vs. Stage I & Stage II)	536	0.487 (0.340-0.693)	<0.001
Histologic grade (G3&G4 vs. G1&G2)	531	0.610 (0.432-0.859)	0.005
Laterality (Right vs. Left)	538	1.368 (0.974-1.923)	0.071

These results suggest that reduced LAMP1 expression may be linked to advanced tumor progression in ccRCC.

### 3.3 LAMP1 expression is associated with a variety of immune cell infiltration

The expression of LAMP1 mRNA and infiltration of 28 immune cell types in ccRCC were analyzed by ssGSEA method. The correlation between immune cell infiltration and LAMP1 mRNA expression was depicted in **Supplementary Figure S2A**. The results revealed a positive association between the expression level of LAMP1 mRNA and iDC, Mast cells, DC, Macrophages, Eosinophils, Tgd, Neutrophils, NK cells, Th17 cells, and B cells. Conversely, it exhibited a negative correlation with Cytotoxic cells, Treg cells, T cells (including helper and CD8 subsets), aDCs (activated dendritic cells), as well as T helper cells (**Supplementary Figures S2B–I**; P<0.01).

### 3.4 The prognostic value of LAMP1 expression in ccRCC patients

**Figure 3:**
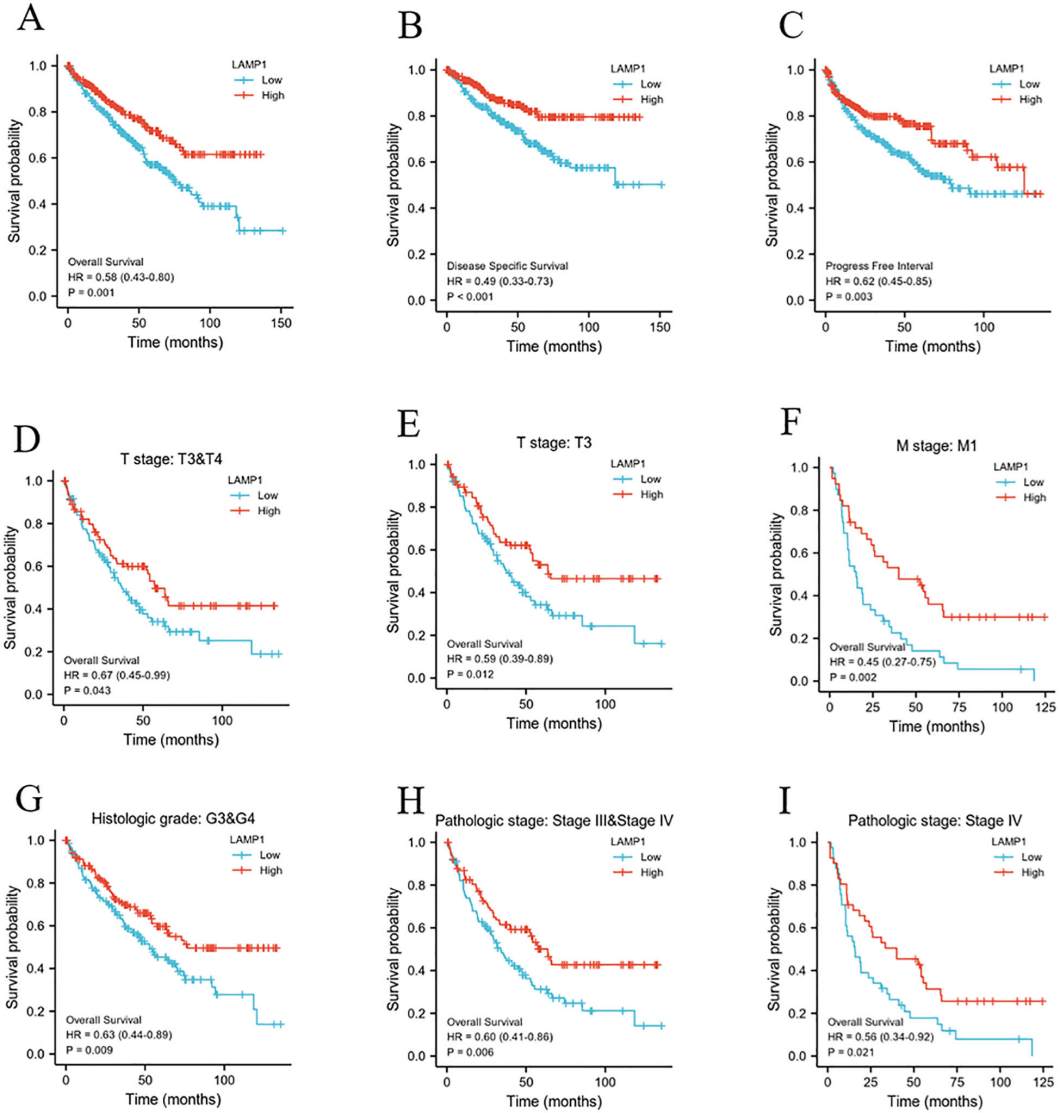
The relationship between LAMP1 expression level and patients’ prognosis and survival. **(A-C)** low LAMP1 expression was associated with worse OS, DSS, and PFI in patients. **(D-I)** OS survival curve of ccRCC patients with T3&4, T3, M1, G3&4, Stage III&IV, and Stage IV subgroups in LAMP1 high and low groups.

The 10-year overall survival rate of patients with low LAMP1 expression was significantly lower compared to those with high LAMP1 expression (**Figure 3A**, P<0.01). Furthermore, the disease-specific survival (DSS) and progression-free interval (PFI) were lower in the group with low LAMP1 expression compared to the group with high LAMP1 expression (**Figures 3B, C**, P<0.01). Subgroup analysis of ccRCC patients revealed that those with low LAMP1 expression had poorer overall survival in Stage T3&4, T3, M1, G3&4, Stage III&IV, and Stage IV (**Figures 3D–I**, P< 0.05). Univariate logistic regression analysis demonstrated that low LAMP1 expression was associated with shorter overall survival[hazard ratio (HR): 0.583; 95% confidence interval (CI): 0.428-0.795; P<0.001] (**Table 2**). To further identify factors related to survival outcomes, multivariate Cox regression analysis was performed considering variables such as T stage, N stage, M stage, pathological stage, histological grade, age and gender; results are presented in **Table 2**.

**Table 2:** Univariate regression and multivariate survival (overall survival) studies of prognostic variables in ccRCC patients.

Characteristics	Total (N)	Univariate analysis		Multivariate analysis	
		Hazard ratio (95% CI)	*P* value	Hazard ratio (95% CI)	*P* value
T stage	539				
T1&T2	349	Reference			
T3&T4	190	3.228 (2.382-4.374)	<0.001	1.915 (0.831-4.414)	0.127
N stage	257				
N0	241	Reference			
N1	16	3.453 (1.832-6.508)	<0.001	1.579 (0.785-3.179)	0.200
M stage	506				
M0	428	Reference			
M1	78	4.389 (3.212-5.999)	<0.001	2.734 (1.608-4.648)	<0.001
Pathologic stage	536				
Stage I & Stage II	331	Reference			
Stage III & Stage IV	205	3.946 (2.872-5.423)	<0.001	1.099 (0.430-2.810)	0.844
Age	539				
<=60	269	Reference			
>60	270	1.765 (1.298-2.398)	<0.001	1.620 (1.057-2.484)	0.027
Gender	539				
Female	186	Reference			
Male	353	0.930 (0.682-1.268)	0.648		
Histologic grade	531				
G1&G2	249	Reference			
G3&G4	282	2.702 (1.918-3.807)	<0.001	1.644 (0.999-2.706)	0.050
LAMP1	539				
Low	269	Reference			
High	270	0.583 (0.428-0.795)	<0.001	0.581 (0.374-0.902)	0.015

### 3.5 A nomogram based on the results of multi-factor analysis

**Figure 4:**
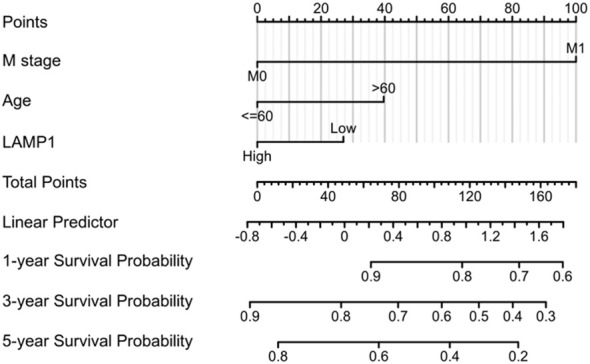
A nomogram predicting 1 -, 3 -, and 5-year survival in ccRCC patients.

To establish a quantitative prognostic tool suitable for clinical application, we developed a nomogram (**Figure 4**) by integrating LAMP1 expression levels with independent clinical risk factors in ccRCC patients.

### 3.6 LAMP1 protein expressed at a low level in ccRCC patients and is associated with poor prognosis

**Figure 5:**
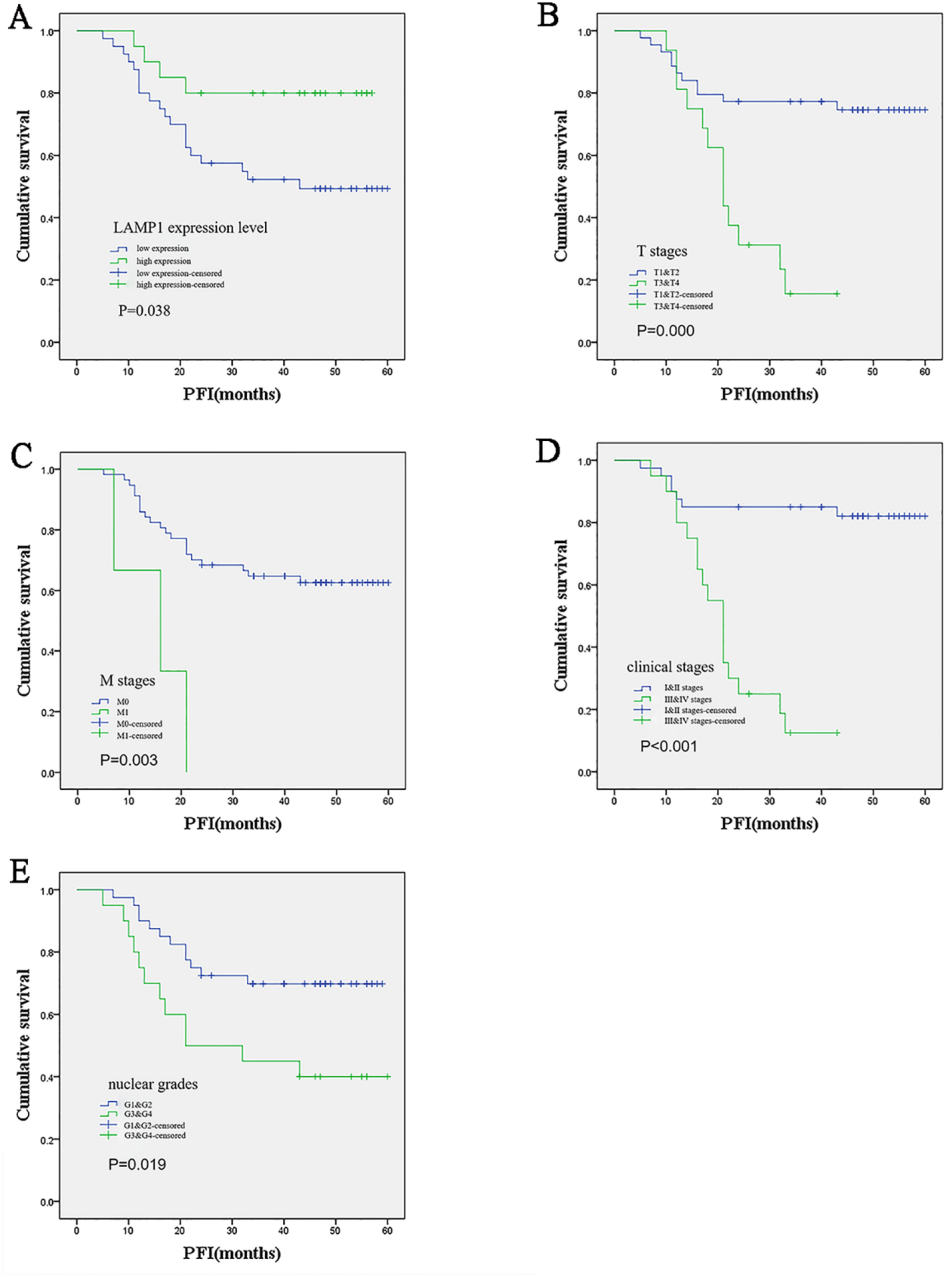
Kaplan-Meier survival analysis of the relationship between prognosis and different clinical variables in ccRCC patients. **(A)** Patients with low LAMP1 expression in cancer tissue had worse prognosis; **(B-E)** The higher the T and M stage, clinical stage and nuclear grade, the worse the prognosis.

Among the 60 patients enrolled in this study, 42 were male and 18 were female; specifically, 33 patients were younger than 60 years old while the remaining 27 patients were older than or equal to 60 years old. Based on protein detection analysis, low expression of LAMP1 was observed in cancer tissues of 40 patients, whereas high expression of LAMP1 was detected in cancer tissues of the other 20 patients. Notably, none of the included patients exhibited lymph node metastasis; hence N stage assessment was not applicable. The remaining clinical information is presented in **Supplementary Table S2**. Correlation analysis revealed significant associations between LAMP1 expression in cancer tissues and sex(P<0.001), age(P<0.001) and T stage(P=0.039), however, no correlations were observed with M stage, clinical stage, or nuclear grade. Western blot analysis was employed to assess LAMP1 protein expression in cancer tissues and corresponding adjacent tissues from 60 pairs of ccRCC patients. As depicted in **Supplementary Figure S3**, LAMP1 expression was significantly lower in cancer tissues compared to paracancerous tissues(P<0.01). Univariate survival analysis was conducted to investigate the relationship between LAMP1 expression and prognosis among ccRCC patients with a mean follow-up time of (35.93 ± 17.547) months and 7 patient deaths occurring during this period. Due to the limited number of terminal events resulting from the short follow-up time and small sample size, progression-free interval (PFI) was used as an evaluation criterion for patient prognosis instead. In total, disease progression occurred in 24 patients (38.7%). Through analysis, we observed a significant association between lower LAMP1 expression in cancer tissues (P=0.038; **Table 3**; **Figure 5A**) and higher T stage (P<0.001; **Table 3**; **Figure 5B**), M stages (P=0.003; **Table 3**; **Figure 5C**), clinical stages (P<0.001; **Table 3**; **Figure 5D**) as well as histologic grade (P=0.019; **Table 3**; **Figure 5E**). Moreover, patients with lower LAMP1 expression exhibited worse prognosis. Subsequently, the multivariate COX regression model was employed to assess the impact of LAMP1 expression level, T and M stages, clinical stages, and nuclear grades on patient prognosis. The results revealed that LAMP1 expression level (P=0.019; **Table 3**), T stage (P=0.040; **Table 3**), clinical stage (P<0.001; **Table 3**), and histologic grade (P=0.043;**Table 3**) independently influenced the prognosis of ccRCC patients.

**Table 3:** Univariate and multivariate COX regression analysis of 60 clinical ccRCC patients.

Variables	Univariate analysis	Multivariate analysis	
	P value	95% CI	P value
M stage	0.003		
LAMP1 expression level	0.038	0.058-0.773	0.019
T stage	<0.001	1.062-14.896	0.040
Clinical stage	<0.001	0.10-0.189	<0.001
Histologic grade	0.019	0.180-0.972	0.043

### 3.7 Overexpression of LAMP1 inhibits the proliferation and migration of renal carcinoma cells

**Figure 6:**
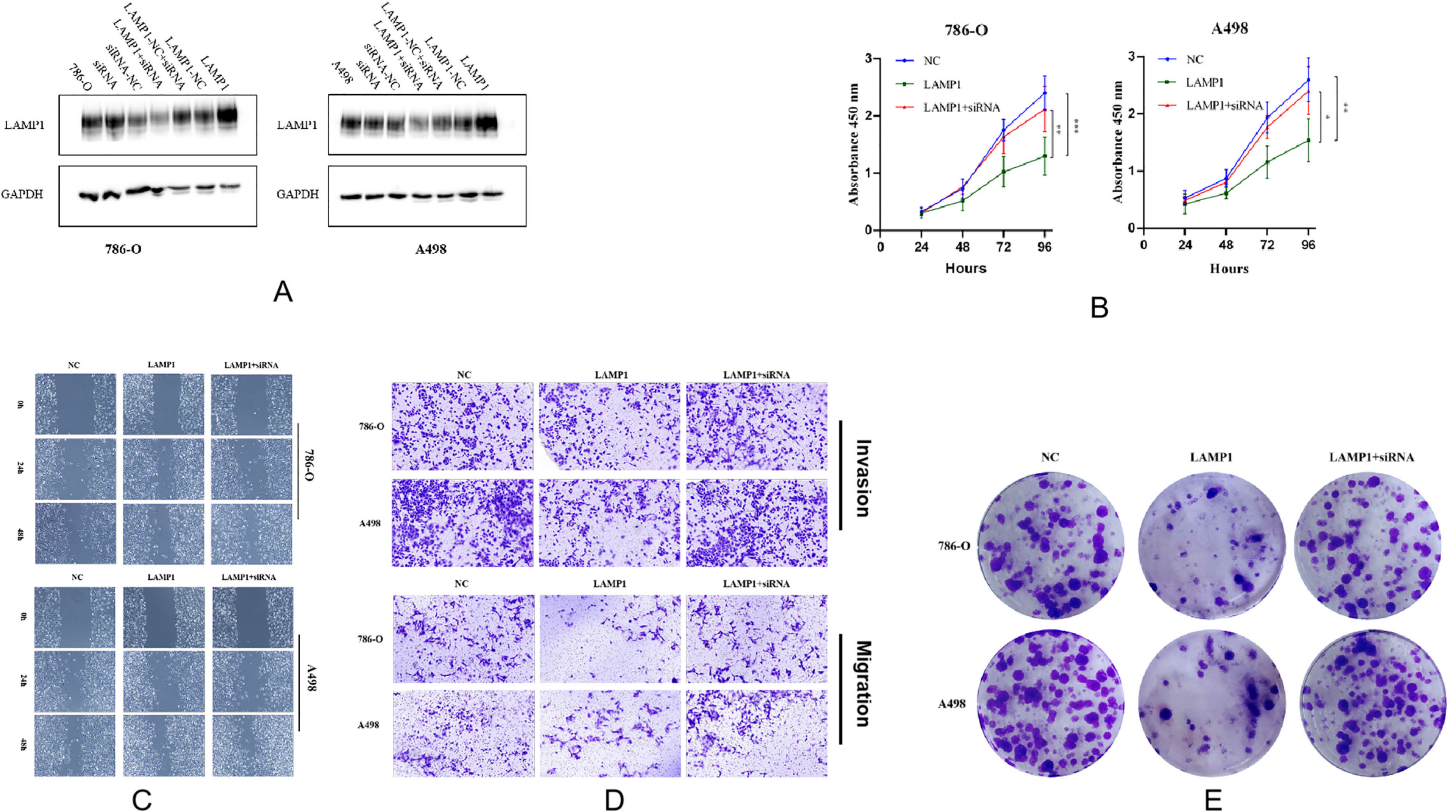
**(A)** The protein detection results after overexpressing LAMP1 gene, knocking down LAMP1 gene, and knocking down LAMP1 gene on the basis of overexpression in 786-O and A498 cells. **(B)** The proliferation curves of 786-O and A498 cells (*P<0.05, **P<0.01, ***P<0.001). **(C)** The effects of LAMP1 overexpression and subsequent knockdown on the migration of 786-O and A498 cells in the wound healing assay. **(D)** The upregulation of LAMP1 levels significantly inhibits the migration and invasion of renal cancer cells *in vitro*. Transwell assay was performed to analyze the effects of upregulation and subsequent downregulation of LAMP1 levels on the migration and invasion of 786-O and A498 cells. **(E)** The effects of LAMP1 overexpression and subsequent knockdown on the colony formation of 786-O and A498 cells.

Virus transfection was performed on 786-O and A498 cells, and Western blot analysis revealed a significant increase in LAMP1 expression in both cell lines. However, siRNA knockdown did not result in a significant decrease in LAMP1 expression, which might be related to the low expression of LAMP1 itself in 786-O and A498 cells, but when conducting the siRNA knockdown experiment based on overexpression of LAMP1 in 786-O cells, we observed that LAMP1 expression returned to normal levels comparable to those of control 786-O cells (**Figure 6A**). The change in cell numbers between the LAMP1 overexpression group and the control group was monitored for three consecutive days, allowing us to construct a proliferation curve. Our results demonstrated that cells with LAMP1 overexpression exhibited slower or nearly negligible rates of proliferation compared to the control group, indicating an inhibitory effect of LAMP1 overexpression on cellular proliferation. Remarkably, upon additional knockdown of LAMP1 using specific siRNAs within the overexpression group, we observed enhanced cell proliferation capacity which closely resembled that of normal proliferating cells (**Figure 6B**). After 0h, 24h, and 48h of microscopic observation of cells in the LAMP1 overexpression group and the control group following scratches, it was observed that the migratory capacity of cells in the LAMP1 overexpression group was diminished compared to that in the control group. Conversely, cell migration ability was enhanced in the rescue experiment (**Figure 6C**). Transwell results further substantiated a significant reduction in the number of LAMP1-overexpressing cells penetrating through the filter membrane compared to the control group. Additionally, when a layer of matrix glue was added to simulate interstitial tissue on the filter membrane, it was found that LAMP1-overexpressing cells exhibited significantly weakened penetration ability; however, this impairment was restored to normal levels during rescue experiments (**Figure 6D**). Following a 14-day cell culture period, both 786-O and A498 cells demonstrated significantly reduced colony formation capability within the LAMP1 overexpression group as opposed to controls. Remarkably though, subsequent rescue experiments revealed recovery of cell colony formation ability compared with previous observations (**Figure 6E**).

**Figure 7:**
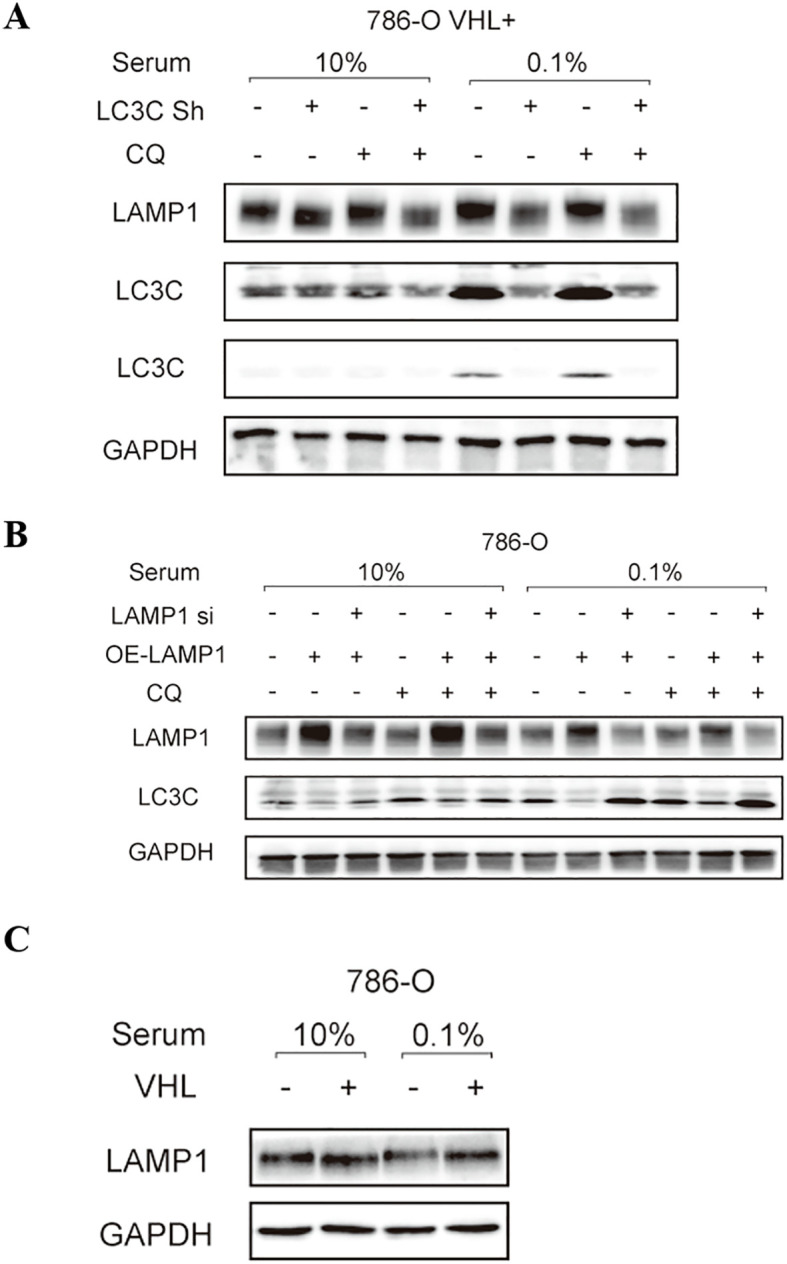
The relationship between LAMP1 and autophagy, LC3C and VHL. **(A)** knockdown LC3C gene LAMP1 expression decreased; **(B)** LC3C expression of overexpressed LAMP1 gene was decreased; **(C)** LAMP1 expression was increased in VHL(+)786-O cells.

The expression of LAMP1 protein in 786-O cells remained unchanged under different serum concentrations, whereas LC3C expression was upregulated under low serum conditions. Upon inhibition of autophagy by adding CQ, no significant alteration was observed in LAMP1 expression, while LC3C expression showed a marked increase. Knockdown of the LC3C gene resulted in reduced levels of LAMP1 expression. Conversely, overexpression of the LAMP1 gene led to decreased LC3C expression (**Figures 7A, B**). Additionally, we observed higher levels of LAMP1 expression in 786-O VHL(+) cells compared to VHL(-) cells (**Figure 7C**).

## 4 Discussion

The discovery of Lysosomal membrane-associated protein 1 (LAMP1) dates back to 1985 when it was first reported by Chen et al. (14) and Lewis et al. (15). Its primary function involves the prevention of lysosomal degradation by autohydrolases through the formation of a highly glycosylated region on the lysosome surface, thereby ensuring proper lysosomal functionality (16). Extensive research has revealed that LAMP1 is implicated in various human diseases, with increasing attention being paid to its role in tumorigenesis. Notably, LAMP1 exhibits diverse functions across different tumor types.

LAMP1 is implicated in tumor progression. Studies have demonstrated that in the ovarian cancer cell line OVCAR3, LAMP1 is upregulated 1.84 times after 24 hours of epidermal growth factor (EGF) treatment but downregulated after 48 hours of exposure. This indicates that LAMP1 may serve as a predictive marker for EGF-induced cell overproliferation (17). In high-grade gliomas (HGG), the cytoplasm and blood vessels of glioblastoma exhibit notable expression of the LAMP1 gene, with significantly higher transcriptional activity compared to normal brain tissue. Immunohistochemistry analysis revealed an increase in both the percentage of LAMP1-positive tumor cells and staining intensity with increasing tumor grade (18). In laryngeal squamous cell carcinoma (LSCC), LAMP1 expression levels are elevated in comparison to normal tissues, showing statistically significant associations with lymph node metastasis and TNM staging (19). Similarly, esophageal squamous cell carcinoma (ESCC) demonstrates a positive correlation between LAMP1 expression levels and TNM staging; higher TNM stages correspond to increased expression levels of LAMP1 (20).

LAMP1 expression has been implicated in prognosis across various tumor types. In diffuse large B-cell lymphoma (DLBCL), the combination of patient LAMP1 expression and survival data demonstrated a correlation between high LAMP1 expression and poor prognosis (21). Furthermore, analysis using a multivariate COX regression model and Kaplan-Meier survival curves in breast cancer revealed that elevated LAMP1 expression is an independent prognostic factor affecting overall patient survival (22). Similarly, overexpression of LAMP1 in epithelial ovarian cancer serves as an indicator of unfavorable prognosis for patients (23). However, contrary to these findings, pancreatic cancer patients with high mRNA levels of LAMP1 exhibited significantly prolonged survival after tumor resection compared to those with low to moderate mRNA expression levels (24). Our database-based survival analysis results indicated that ccRCC patients with high LAMP1 expression had significantly longer overall survival (OS), disease-specific survival (DSS), and progression-free interval (PFI) than those with low LAMP1 expression. Long-term follow-up data from 60 patients further demonstrated shortened PFI specifically among individuals with low LAMP1 expression compared to their counterparts exhibiting high LAMP1 levels. Clearly, the impact of LAMP1 on patient survival varies depending on the specific tumor type, potentially reflecting its distinct roles within different malignancies.

Utilizing shRNA to downregulate LAMP1 in B16F10 mouse melanoma cells can attenuate the induction of matrix metalloproteinase (MMP9) expression by the p38 MAPK signaling pathway activated by galectin-3 and polyN-acetyllactosamine (polyLacNAc) (25). Subsequent investigations have demonstrated that LAMP1 interacts with galectin-3 on the lung through polyLacNAc, which is implicated in melanoma lung metastasis (26). In metastatic melanoma cells A2058, indirect immunofluorescence staining has revealed clustering of LAMP molecules at the cell periphery, suggesting potential involvement of LAMP1 in cell adhesion (27). In non-small cell lung cancer (NSCLC), miR-373 expression is downregulated, significantly impacting proliferation, migration, and invasion of lung cancer cell lines upon its upregulation. As a downstream target of miR-373, LAMP1 also inhibits tumor migration and invasion when knocked out. Furthermore, overexpression of LAMP1 correlates with lymph node metastasis in NSCLC (28). Similarly, in prostate cancer, miR-320a functions as a tumor suppressor miRNA. To investigate the downstream targets of miR-320a, researchers identified target genes through whole-genome gene expression analysis, including the LAMP1 gene. Overexpression of LAMP1 was observed in clinical specimens of prostate cancer and metastatic prostate cancer. Silencing LAMP1 using siRNA significantly inhibits the proliferation, migration, and invasion of prostate cancer cells (29). Colon cancer cells with strong metastatic ability express higher levels of LAMP1 protein on their surface, and it has been demonstrated that LAMP1 binds to E-selectin through terminal sialylated Lewis oligosaccharide -X (SLeX), promoting colon cancer cell metastasis (30). Experimental research revealed that overexpression of the LAMP1 gene effectively reduces the proliferation ability of 786-O and A498 cells; however, when overexpression is followed by knockdown restoration restores its ability. In migration and invasion experiments, cell migration ability significantly weakens after LAMP1 overexpression along with decreased penetration into matrix glue. Similarly, these abilities are restored after LAMP1 overexpression. Furthermore, our cell cloning experiments showed a significant reduction in colony formation after LAMP1 overexpression but restoration upon returning to normal levels of expression. These results indicate that LAMP1 can inhibit the proliferation, migration and invasion of tumor cells. These results indicate that LAMP1 can inhibit tumor cell proliferation, migration, and invasion. This contradicts its role in other tumors suggesting alternative mechanisms may be at play.

LAMP1 exhibits tumor-specific variations and distinct mechanisms of action, even within the same tumor type. Currently, there is a lack of comprehensive studies elucidating the precise mechanism underlying LAMP1’s involvement in tumorigenesis. However, experimental evidence suggests that LAMP1 can impede renal cancer cell line proliferation, migration, and invasion. It is plausible that LAMP1 may be associated with tumor autophagy and VHL gene regulation; nevertheless, its specific mode of action in ccRCC remains elusive. Nonetheless, it is evident that LAMP1 exerts an inhibitory effect on ccRCC progression, as downregulation of LAMP1 expression correlates with poorer patient prognosis.

Autophagy is a form of lysosomal-mediated cell degradation. Although the basal level of autophagy in most tissues is low, it can be significantly induced by stress conditions, with hunger being the most extensively studied one, which is tightly regulated by mTOR and AMP kinase pathways (31, 32). Due to the rapid proliferation of cancer cells and alterations in glycolytic metabolism, cancer cells have higher metabolic demands compared to normal cells (33), necessitating the involvement of autophagy (34). In cervical cancer, transcriptome sequencing and bioinformatics analysis revealed that LAMP1 acts as a downstream regulator of autophagy and participates in HPV-16E6/E7-mediated regulation of autophagy in cervical cancer cells (35). A study on pancreatic cancer demonstrated that ubiquitin-like protein UBL4A can modulate the proliferation and metastasis of pancreatic ductal adenocarcinoma (PDAC) through inhibition of autophagy. Additionally, LAMP1 was identified as a direct target responsible for UBL4A-induced tumor suppression and autophagy inhibition. Disruption of lysosomal function by LAMP1 leads to impaired degradation of autophagosomes (36). Similarly, research on prostate cancer revealed that Abrus agglutinin (AGG) induces lipophagy - a selective pathway involving autophagic breakdown of lipids - resulting in the production of free fatty acids (FFAs) and triggering senescence in PC3 cells. Deacetylation process targeting LAMP1 promotes AGG-induced lipolysis-dependent generation FFAs thereby promoting senescence specifically in prostate cancer. It becomes evident that LAMP1 not only serves as a downstream target for miR-320a but also influences prostate cancer progression through modulation cell-autonomous mechanisms governing cellular self-degradation processes such as autophag (37).

Renal cell carcinoma (RCC) is a highly heterogeneous tumor, and the current primary focus lies in investigating the molecular mechanisms underlying tumorigenesis and development, as well as implementing precise targeted therapy. Currently, classic medications for advanced RCC targeted therapy involve the development of tumor inhibitors that target the VEGF/VEGFR signaling pathway and the PI3K/Akt/mTOR signaling pathway. Although LAMP1’s mechanism has been reported in other tumors, its role in ccRCC remains unclear. Through further experimentation, our aim is to elucidate the specific molecular mechanisms of LAMP1, thereby providing novel insights for targeted therapy of RCC.

In our experiment, we induced cellular autophagy by subjecting the cells to starvation conditions. Surprisingly, we observed that the expression of LAMP1 remained largely unchanged during starvation. Subsequently, when we blocked the autophagy process using CQ, there was still no significant alteration in LAMP1 expression. These findings suggest that LAMP1 may primarily function in the late stages of autophagy and might not be involved in its initiation or fusion between autophagosomes and lysosomes. LC3C, a pivotal gene in autophagy, exhibits increased expression concomitant with enhanced autophagy and significantly upregulates when the autophagy process is inhibited. Knockdown of LC3C resulted in decreased expression of LAMP1, suggesting that LAMP1 may function as a downstream target of LC3C. However, overexpression of the LAMP1 gene led to a decrease in the expression of LC3C instead, which was restored upon subsequent knockdown of LAMP1. This suggests that LAMP1 might exert a negative feedback mechanism contributing to the downregulation of the LC3C gene expression. Nevertheless, at present, we have only preliminarily demonstrated the correlation between LAMP1 and the autophagy-related gene LC3C at the protein expression level. Due to the lack of in-depth research, the specific interaction between LAMP1 and LC3C remains unclear, and further investigations are required to elucidate the precise underlying mechanisms.

VHL gene is the most frequently mutated gene in ccRCC, exerting inhibitory effects on tumor growth. Several studies have demonstrated that VHL plays a crucial role in maintaining lysosomal integrity. Cells lacking VHL exhibit unstable lysosomal membranes and are susceptible to degradation (38). In an experiment investigating the specific cytotoxicity of drug STF-62247 towards VHL-deficient cells, it was observed that VHL-rich cells can overcome the pressure induced by drug STF by enhancing the mobility of LAMP1 towards the nucleus by monitoring the position of lysosomes in cells (39).This observation further suggests a potential association between VHL and LAMP1. Previous research has also revealed a positive regulatory effect of VHL on LC3C, leading us to speculate that LAMP1 may serve as a downstream target of LC3C based on experimental findings. Further analysis confirmed higher expression levels of LAMP1 in VHL(+)786-O cells. Consequently, we propose the existence of a regulatory pathway involving VHL-LC3C-LAMP1 in ccRCC. However, due to the limitations of our current study, this potential pathway still awaits further experimental validation. Yet, we are confident that there are interactions among LAMP1, LC3C, and the VHL gene. Future experimental research will definitely help us clarify the detailed mechanisms underlying their interactions.

## 5 Conclusions

In summary, LAMP1 is downregulated in ccRCC, and its decreased expression is frequently associated with unfavorable prognosis in patients. LAMP1 exerts anti-tumor effects by suppressing the proliferation, migration, and invasion of renal cancer cells. Moreover, we propose that this anti-tumor effect is mediated through LC3C-induced tumor autophagy and is linked to VHL. Our study highlights the potential of LAMP1 as a novel biomarker for diagnosing ccRCC patients and predicting their prognosis, while also providing valuable insights into the underlying molecular mechanisms that could guide targeted therapeutic strategies for renal cancer.

## Data Availability

The original contributions presented in the study are included in the article/**Supplementary Files**, further inquiries can be directed to the corresponding author/s.

## References

[1] Bray F Ferlay J Soerjomataram I Siegel RL Torre LA Jemal A. Global cancer statistics 2018: GLOBOCAN estimates of incidence and mortality worldwide for 36 cancers in 185 countries. CA Cancer J Clin. (2018) 68:394–424. doi: 10.3322/caac.21492 30207593

[2] Shuch B Amin A Armstrong AJ Eble JN Ficarra V Lopez-Beltran A. Understanding pathologic variants of renal cell carcinoma: distilling therapeutic opportunities from biologic complexity. Eur Urol. (2015) 67:85–97. doi: 10.1016/j.eururo.2014.04.029 24857407

[3] Welch HG Skinner JS Schroeck FR Zhou W Black WC. Regional variation of computed tomographic imaging in the United States and the risk of nephrectomy. JAMA Intern Med. (2018) 178:221–7. doi: 10.1001/jamainternmed.2017.7508 PMC5838611 29279887

[4] Znaor A Lortet-Tieulent J Laversanne M Jemal A Bray F. International variations and trends in renal cell carcinoma incidence and mortality. Eur Urol. (2015) 67:519–30. doi: 10.1016/j.eururo.2014.10.002 25449206

[5] Dabestani S Thorstenson A Lindblad P Harmenberg U Ljungberg B Lundstam S. Renal cell carcinoma recurrences and metastases in primary non-metastatic patients: a population-based study. World J Urol. (2016) 34:1081–6. doi: 10.1007/s00345-016-1773-y 26847337

[6] Rao A Wiggins C Lauer RC. Survival outcomes for advanced kidney cancer patients in the era of targeted therapies. Ann Transl Med. (2018) 6:165. doi: 10.21037/atm.2018.04.44 29911113 PMC5985277

[7] Winchester BG. Lysosomal membrane proteins. Eur J Paediatr Neurol. (2001) 5 Suppl A:11–9. doi: 10.1053/ejpn.2000.0428 11588980

[8] Wilke S Krausze J Bussow K. Crystal structure of the conserved domain of the DC lysosomal associated membrane protein: implications for the lysosomal glycocalyx. BMC Biol. (2012) 10:62. doi: 10.1186/1741-7007-10-62 22809326 PMC3409847

[9] White E. The role for autophagy in cancer. J Clin Invest. (2015) 125:42–6. doi: 10.1172/JCI73941 PMC4382247 25654549

[10] Mikhaylova O Stratton Y Hall D Kellner E Ehmer B Drew AF. VHL-regulated MiR-204 suppresses tumor growth through inhibition of LC3B-mediated autophagy in renal clear cell carcinoma. Cancer Cell. (2012) 21:532–46. doi: 10.1016/j.ccr.2012.02.019 PMC3331999 22516261

[11] Hall DP Cost NG Hegde S Kellner E Mikhaylova O Stratton Y. TRPM3 and miR-204 establish a regulatory circuit that controls oncogenic autophagy in clear cell renal cell carcinoma. Cancer Cell. (2014) 26:738–53. doi: 10.1016/j.ccell.2014.09.015 PMC4269832 25517751

[12] Bell ES Coelho PP Ratcliffe CDH Rajadurai CV Peschard P Vaillancourt R. LC3C-mediated autophagy selectively regulates the met RTK and HGF-stimulated migration and invasion. Cell Rep. (2019) 29:4053–68 e6. doi: 10.1016/j.celrep.2019.11.063 31851933

[13] Li T Fan J Wang B Traugh N Chen Q Liu JS. TIMER: A web server for comprehensive analysis of tumor-infiltrating immune cells. Cancer Res. (2017) 77:e108–e10. doi: 10.1158/0008-5472.CAN-17-0307 PMC6042652 29092952

[14] Chen JW Murphy TL Willingham MC Pastan I August JT. Identification of two lysosomal membrane glycoproteins. J Cell Biol. (1985) 101:85–95. doi: 10.1083/jcb.101.1.85 2409098 PMC2113627

[15] Lewis V Green SA Marsh M Vihko P Helenius A Mellman I. Glycoproteins of the lysosomal membrane. J Cell Biol. (1985) 100:1839–47. doi: 10.1083/jcb.100.6.1839 PMC2113609 3922993

[16] Saftig P Klumperman J. Lysosome biogenesis and lysosomal membrane proteins: trafficking meets function. Nat Rev Mol Cell Biol. (2009) 10:623–35. doi: 10.1038/nrm2745 19672277

[17] Marzinke MA Choi CH Chen L Shih Ie M Chan DW Zhang H. Proteomic analysis of temporally stimulated ovarian cancer cells for biomarker discovery. Mol Cell Proteomics. (2013) 12:356–68. doi: 10.1074/mcp.M112.019521 PMC3567859 23172893

[18] Sarafian VS Koev I Mehterov N Kazakova M Dangalov K. LAMP-1 gene is overexpressed in high grade glioma. APMIS. (2018) 126:657–62. doi: 10.1111/apm.2018.126.issue-8 29920782

[19] Lu M Zhu H Wang X Zhang D Xiong L Zhu J. LAMP1 expression is associated with Malignant behaviours and predicts unfavourable prognosis in laryngeal squamous cell carcinoma. Pathology. (2016) 48:684–90. doi: 10.1016/j.pathol.2016.08.001 27788920

[20] Huang J Li L Liu J Yu J Wu X Xu Y. Altered expression of lysosomal associated membrane protein 1 in esophageal squamous cell carcinoma. Pathol Res Pract. (2017) 213:938–42. doi: 10.1016/j.prp.2017.05.008 28687162

[21] Dang Q Zhou H Qian J Yang L Huang J Zhang Y. LAMP1 overexpression predicts for poor prognosis in diffuse large B-cell lymphoma. Clin Lymph Myeloma Leuk. (2018) 18:749–54. doi: 10.1016/j.clml.2018.07.288 30082222

[22] Wang Q Yao J Jin Q Wang X Zhu H Huang F. LAMP1 expression is associated with poor prognosis in breast cancer. Oncol Lett. (2017) 14:4729–35. doi: 10.3892/ol.2017.6757 PMC5649640 29085473

[23] Xu Y Cao X Zhang S Zhang Y Shen Z. High expression of LAMP1 as a prognostic marker in patients with epithelial ovarian cancer. Int J Clin Exp Pathol. (2017) 10:9104–11. PMC6965383 31966783

[24] Kunzli BM Berberat PO Zhu ZW Martignoni M Kleeff J Tempia-Caliera AA. Influences of the lysosomal associated membrane proteins (Lamp-1, Lamp-2) and Mac-2 binding protein (Mac-2-BP) on the prognosis of pancreatic carcinoma. Cancer. (2002) 94:228–39. doi: 10.1002/cncr.10162 11815981

[25] Dange MC Agarwal AK Kalraiya RD. Extracellular galectin-3 induces MMP9 expression by activating p38 MAPK pathway via lysosome-associated membrane protein-1 (LAMP1). Mol Cell Biochem. (2015) 404:79–86. doi: 10.1007/s11010-015-2367-5 25739356

[26] Agarwal AK Srinivasan N Godbole R More SK Budnar S Gude RP. Role of tumor cell surface lysosome-associated membrane protein-1 (LAMP1) and its associated carbohydrates in lung metastasis. J Cancer Res Clin Oncol. (2015) 141:1563–74. doi: 10.1007/s00432-015-1917-2 PMC11823972 25614122

[27] Sarafian V Jadot M Foidart JM Letesson JJ Van den Brule F Castronovo V. Expression of Lamp-1 and Lamp-2 and their interactions with galectin-3 in human tumor cells. Int J Cancer. (1998) 75:105–11. doi: 10.1002/(SICI)1097-0215(19980105)75:1<105::AID-IJC16>3.0.CO;2-F 9426697

[28] Seol HS Akiyama Y Shimada S Lee HJ Kim TI Chun SM. Epigenetic silencing of microRNA-373 to epithelial-mesenchymal transition in non-small cell lung cancer through IRAK2 and LAMP1 axes. Cancer Lett. (2014) 353:232–41. doi: 10.1016/j.canlet.2014.07.019 PMC7707239 25063738

[29] Okato A Goto Y Kurozumi A Kato M Kojima S Matsushita R. Direct regulation of LAMP1 by tumor-suppressive microRNA-320a in prostate cancer. Int J Oncol. (2016) 49:111–22. doi: 10.3892/ijo.2016.3522 PMC4902064 27212625

[30] Saitoh O Wang WC Lotan R Fukuda M. Differential glycosylation and cell surface expression of lysosomal membrane glycoproteins in sublines of a human colon cancer exhibiting distinct metastatic potentials. J Biol Chem. (1992) 267:5700–11. doi: 10.1016/S0021-9258(18)42823-2 1544942

[31] Mancias JD Kimmelman AC. Mechanisms of selective autophagy in normal physiology and cancer. J Mol Biol. (2016) 428:1659–80. doi: 10.1016/j.jmb.2016.02.027 PMC4846542 26953261

[32] Khaminets A Behl C Dikic I. Ubiquitin-dependent and independent signals in selective autophagy. Trends Cell Biol. (2016) 26:6–16. doi: 10.1016/j.tcb.2015.08.010 26437584

[33] White E DiPaola RS. The double-edged sword of autophagy modulation in cancer. Clin Cancer Res. (2009) 15:5308–16. doi: 10.1158/1078-0432.CCR-07-5023 PMC2737083 19706824

[34] Amaravadi RK Lippincott-Schwartz J Yin XM Weiss WA Takebe N Timmer W. Principles and current strategies for targeting autophagy for cancer treatment. Clin Cancer Res. (2011) 17:654–66. doi: 10.1158/1078-0432.CCR-10-2634 PMC3075808 21325294

[35] Tingting C Shizhou Y Songfa Z Junfen X Weiguo L Xiaodong C. Human papillomavirus 16E6/E7 activates autophagy via Atg9B and LAMP1 in cervical cancer cells. Cancer Med. (2019) 8:4404–16. doi: 10.1002/cam4.2351 PMC6675746 31215164

[36] Chen H Li L Hu J Zhao Z Ji L Cheng C. UBL4A inhibits autophagy-mediated proliferation and metastasis of pancreatic ductal adenocarcinoma via targeting LAMP1. J Exp Clin Cancer Res. (2019) 38:297. doi: 10.1186/s13046-019-1278-9 31288830 PMC6617940

[37] Panda PK Patra S Naik PP Praharaj PP Mukhopadhyay S Meher BR. Deacetylation of LAMP1 drives lipophagy-dependent generation of free fatty acids by Abrus agglutinin to promote senescence in prostate cancer. J Cell Physiol. (2020) 235:2776–91. doi: 10.1002/jcp.v235.3 31544977

[38] Bouhamdani N Comeau D Cormier K Turcotte S. STF-62247 accumulates in lysosomes and blocks late stages of autophagy to selectively target von Hippel-Lindau-inactivated cells. Am J Physiol Cell Physiol. (2019) 316:C605–C20. doi: 10.1152/ajpcell.00483.2018 30758995

[39] Bouhamdani N Comeau D Coholan A Cormier K Turcotte S. Targeting lysosome function causes selective cytotoxicity in VHL-inactivated renal cell carcinomas. Carcinogenesis. (2020) 41:828–40. doi: 10.1093/carcin/bgz161 PMC7351131 31556451

