# Supplementary material for: Research on the effect of LAMP1 in the development and progression of ccRCC and its potential mechanism with LC3C-mediated autophagy
Source: Front Immunol. 2024 Nov 28;15:1494005. doi: 10.3389/fimmu.2024.1494005 (PMC11634794; doi:10.3389/fimmu.2024.1494005)
Supplement: Supplementary file 1 [file DataSheet1.docx]

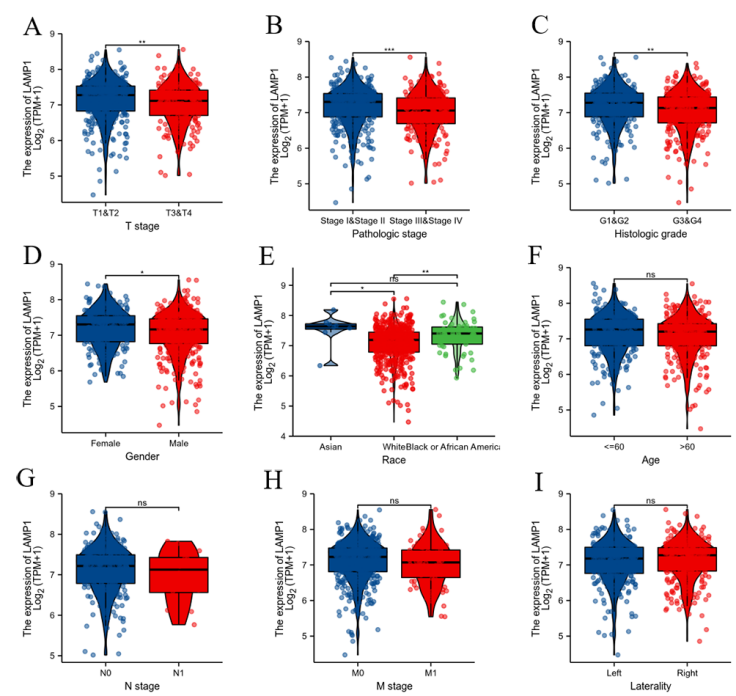


Supplement Figure1. The relationship between LAMP1 expression level and clinicopathological features included **(A)** T stage; **(B)** clinicopathological grade; **(C)** Nuclear classification; **(D)** Gender; **(E)** Ethnicity; **(F)** Age; **(G)** N staging; **(H)** M staging; **(I)** Tumor location (*P<0.05, **P<0.01, ***P<0.001)


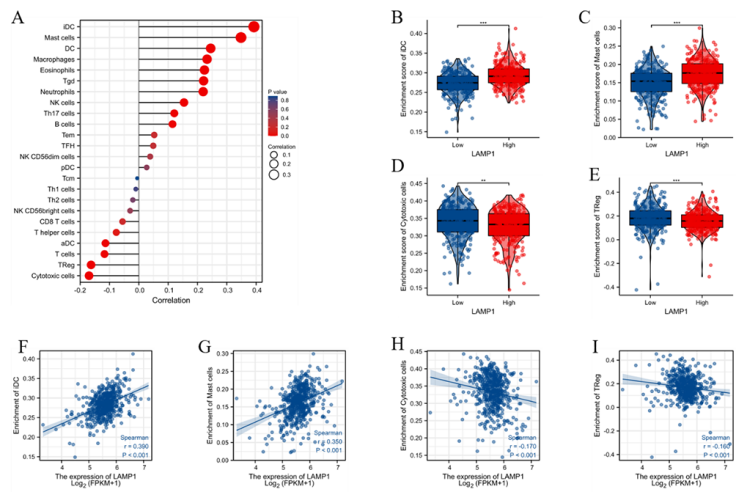


Supplement Figure2. LAMP1 expression level is associated with immune infiltration in the tumor microenvironment. **(A)** The relationship between the relative abundance of 24 types of immune cells and LAMP1 expression levels; **(B-I)** Histogram and scatter plot showed the relationship between LAMP1 expression level and infiltration level of iDC, Mast cells, Cytotoxics cells, and Treg cells (**P<0.01, ***P<0.001)


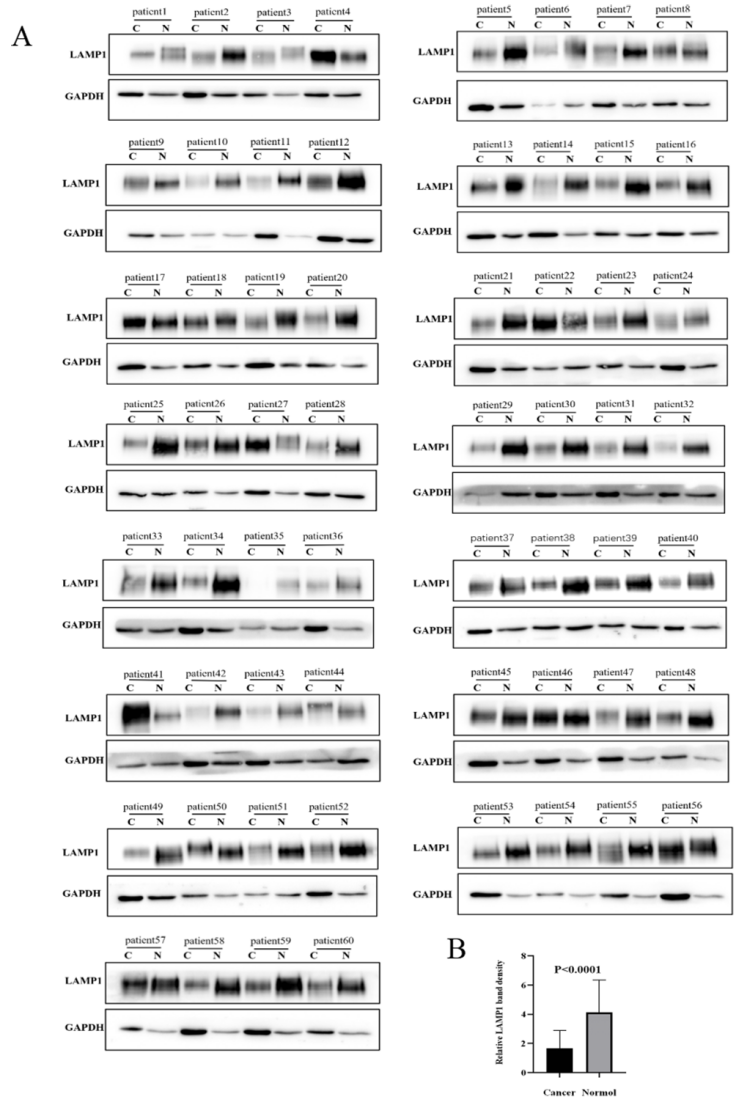


Supplement Figure3. Protein detection of cancer and adjacent tissues in 60 ccRCC patients **( A)** and quantitative analysis**(B)**
