# Supplementary material for: Research on the effect of LAMP1 in the development and progression of ccRCC and its potential mechanism with LC3C-mediated autophagy
Source: Front Immunol. 2024 Nov 28;15:1494005. doi: 10.3389/fimmu.2024.1494005 (PMC11634794; doi:10.3389/fimmu.2024.1494005)
Supplement: Supplementary file 2 [file Table1.docx]

Supplement Table 1: Clinical characteristics of ccRCC patients in the TCGA database (n = 539)

| Characteristic | levels | Overall |
| --- | --- | --- |
| n |  | 539 |
| T stage, n (%) | T1 | 278 (51.6%) |
|  | T2 | 71 (13.2%) |
|  | T3 | 179 (33.2%) |
|  | T4 | 11 (2%) |
| N stage, n (%) | N0 | 241 (93.8%) |
|  | N1 | 16 (6.2%) |
| M stage, n (%) | M0 | 428 (84.6%) |
|  | M1 | 78 (15.4%) |
| Gender, n (%) | Female | 186 (34.5%) |
|  | Male | 353 (65.5%) |
| Pathologic stage, n (%) | Stage I | 272 (50.7%) |
|  | Stage II | 59 (11%) |
|  | Stage III | 123 (22.9%) |
|  | Stage IV | 82 (15.3%) |
| Primary therapy outcome, n (%) | PD | 11 (7.5%) |
|  | SD | 6 (4.1%) |
|  | PR | 2 (1.4%) |
|  | CR | 128 (87.1%) |
| Race, n (%) | Asian | 8 (1.5%) |
|  | Black or African American | 57 (10.7%) |
|  | White | 467 (87.8%) |
| Age, n (%) | <=60 | 269 (49.9%) |
|  | >60 | 270 (50.1%) |
| Histologic grade, n (%) | G1 | 14 (2.6%) |
|  | G2 | 235 (44.3%) |
|  | G3 | 207 (39%) |
|  | G4 | 75 (14.1%) |
| Laterality, n (%) | Left | 252 (46.8%) |
|  | Right | 286 (53.2%) |
| Age, median (IQR) |  | 61 (52, 70) |

Supplement Table 2: Basic information of 60 clinical patients

| Clinical variables | LAMP1 expression level in cancer tissue | | P value |
| --- | --- | --- | --- |
|  | Low expression (n=40) | High expression(n=20) |  |
| Gender |  |  | <0.001 |
| male | 27 | 15 |  |
| femble | 13 | 5 |  |
| age |  |  | <0.001 |
| <60 | 21 | 13 |  |
| >=60 | 19 | 7 |  |
| T stage |  |  | 0.039 |
| T1&T2 | 26 | 18 |  |
| T3&T4 | 14 | 2 |  |
| M stage |  |  | 0.209 |
| M0 | 39 | 18 |  |
| M1 | 1 | 2 |  |
| Clinical stage |  |  | 0.121 |
| Ⅰ & Ⅱ stage | 24 | 16 |  |
| Ⅲ & Ⅳ stage | 16 | 4 |  |
| Histologic grade |  |  | 0.846 |
| G1&G2 | 27 | 13 |  |
| G3&G4 | 13 | 7 |  |
